# Impact on outcomes of measuring lactates prior to ICU in unselected heterogeneous critically ill patients: A propensity score analysis

**DOI:** 10.1371/journal.pone.0277948

**Published:** 2022-11-28

**Authors:** Taro Tamakawa, Hiroshi Endoh, Natuo Kamimura, Kazuki Deuchi, Kei Nishiyama

**Affiliations:** 1 Niigata University Faculty of Medicine, Department of Emergency & Critical Care Medicine, Niigata City, Niigata, Japan; 2 Advanced Emergency and Critical Care Center, Niigata University Medical & Dental Hospital, Niigata City, Niigata, Japan; Clinica Luganese Moncucco, SWITZERLAND

## Abstract

**Background:**

Elevated blood lactate levels were reported as effective predictors of clinical outcome and mortality in ICU. However, there have been no studies simply comparing the timing of measuring lactates before vs. after ICU admission.

**Methods:**

A total of 19,226 patients with transfer time ≤ 24 hr were extracted from the Medical Information Mart for Intensive Care IV database (MIMIC-IV). After 1:1 propensity score matching, the patients were divided into two groups: measuring lactates within 3 hr before (BICU group, n = 4,755) and measuring lactate within 3 hr after ICU admission(AICU group, n = 4,755). The primary and secondary outcomes were hospital mortality, hospital 28-day mortality, ICU mortality, ICU length of stay (LOS), hospital LOS, and restricted mean survival time (RMST).

**Results:**

Hospital, hospital 28-day, and ICU mortality were significantly higher in AICU group (7.0% vs.9.8%, 6.7% vs. 9.4%, and 4.6% vs.6.7%, respectively, *p*<0.001 for all) Hospital LOS and ICU LOS were significantly longer in AICU group (8.4 days vs. 9.0 days and 3.0 days vs. 3.5 days, respectively, *p*<0.001 for both). After adjustment for predefined covariates, a significant association between the timing of measuring lactate and hospital mortality was observed in inverse probability treatment weight (IPTW) multivariate regression, doubly robust multivariate regression, and multivariate regression models (OR, 0.96 [95%CI, 0.95-0.97], OR 0.52 [95%CI, 0.46-0.60], OR 0.66 [95%CI, 0.56-0.78], respectively, p<0.001 for all), indicating the timing as a significant risk-adjusted factor for lower hospital mortality. The difference (BICU-AICU) of RMST at 28- days after ICU admission was 0.531 days (95%CI, 0.002-1.059, p<0.05). Placement of A-line and PA-catheter, administration of intravenous antibiotics, and bolus fluid infusion during the first 24-hr in ICU were significantly more frequent and faster in the BICU vs AICU group (67.6% vs. 51.3% and 126min vs.197min for A-line, 19.6% vs.13.2% and 182min vs. 274min for PA-catheter, 77.5% vs.67.6% and 109min vs.168min for antibiotics, and 57.6% vs.51.6% and 224min vs.278min for bolus fluid infusion, respectively, p<0.001 for all). Additionally, a significant indirect effect was observed in frequency (0.19879 [95% CI, 0.14061-0.25697] p<0.001) and time (0.07714 [95% CI, 0.22600-0.13168], p<0.01) of A-line replacement, frequency of placement of PA-catheter (0.05614 [95% CI, 0.04088-0.07140], p<0.001) and frequency of bolus fluid infusion (0.02193 [95%CI, 0.00303-0.04083], p<0.05).

**Conclusions:**

Measuring lactates within 3 hr prior to ICU might be associated with lower hospital mortality in unselected heterogeneous critically ill patients with transfer time to ICU ≤ 24hr, presumably due to more frequent and faster therapeutic interventions.

## Introduction

An elevated blood lactate level mainly results from anaerobic metabolism caused by tissue hypoxia, accelerated aerobic glycolysis via the Na-K ATPase due to excess β-adrenergic stimulation, or impaired clearance from the liver [1,2]. While blood lactate level is not a direct reflection of tissue perfusion, it can serve as a surrogate marker of poor tissue perfusion [3].

Before now, numerous studies have shown that whether a single static value or serial dynamic index, lactate measurements are effective predictors for severity, prognosis, or mortality in diverse cohorts: septic [4–8], traumatic [9], surgical [10], critically ill [11–13], and pediatric patients [14]; or in diverse locations: prehospital settings[15,16], emergency department (ED) [9,17,18], general ward [4,14], and intensive care unit (ICU) [4–8,11–13].

It is conceivable that elevated blood lactate levels prior to ICU admission may hasten therapeutic interventions, and thereby would improve morbidity or mortality. However, a large number of studies appear to discuss the lactate values alone without considerations of lactate data prior to ICU admission. To our knowledge, there have been no comparative studies of the timing of measuring lactates before and after ICU admission.

Thus, the present study was conducted to simply compare the impacts of measuring lactates within 3 hr before vs. within 3 hr after ICU admission on outcomes in unselected heterogeneous ICU patients.

## Materials and methods

### Data sources

The medical information mart for intensive care (Multiparameter Intelligent Monitoring for Intensive Care IV [MIMIC-IV] version 0.4) is a large, freely available database comprising deidentified health-related data associated with 53,150 patients who stayed in seven ICUs of the Beth Israel Deaconess Medical Center (Boston, MA, USA) between 2008 and 2019 [19, 20]. The use of the MIMIC- IV database was approved for HE after certification of the CITI program by the Massachusetts Institute of Technology (No. 25459972).

### Patient selection

Eligibility criteria included the first ICU admission on a same hospital admission, ICU length of stay (LOS) ≥ 12hr, age ≥18 years, and the time interval between hospital admission and ICU admission (transfer time) ≤ 24 hr.

According to the timing of measuring lactates relevant to ICU admission, the patients were divided into two groups: measuring lactates within 3 hr before (BICU group) vs. measuring lactates within 3 hr after ICU admission (AICU group).

### Variable extraction

The extracted variables for patients were as follows: age, gender, ethnicity, admission type, ICU type, ICU severity scores (the first-day Sequential Organ Failure Assessment [SOFA] score and Simplified Acute Physiology Score[SAPS] II score), Charlson Comorbidity Index (CCI), arterial blood lactate level, the first-day Sepsis-3, the first-day therapeutic interventions (use of mechanical and non-invasive ventilation, vasopressor, or renal replacement therapy [RRT], intravenous [IV] administration of antibiotics, placement of arterial line [A-line], central venous [CV] - catheter or pulmonary artery [PA] - catheter, and bolus fluid infusion), the first-day vital signs (heart rate, mean arterial pressure [MAP], respiratory rate, and SpO_2_), and the first-day laboratory data (hemoglobin, hematocrit, white blood cell [WBC] count, platelet count, sodium, chloride, and potassium level).

The structured query language (SQL) scripts for data extraction are available on the GitHub website (https://github.com/MIT-LCP/mimic-IV).

### Study endpoints

The primary exposure was the timing of measuring lactates relevant to ICU admission (BICU group vs. AICU group). The primary outcome was to examine the difference of hospital mortality between the two groups.

The secondary outcome included differences in ICU mortality, hospital 28-day mortality, hospital LOS, ICU LOS, and restricted mean survival time [21,22] between the two groups.

### Statistical analysis

Descriptive statics were computed for all variables, and normal distribution was assessed using Shapiro-Wilk test. Categorical variables were presented as numbers and percentages (%) and were compared using chi-squared test. Continuous variables were presented as mean±standard deviation (SD) for variables with normal distribution or as median (interquartile range [IQR]) for variables without normal distribution and compared using either Student’s *t*-test or Mann-Whitney *U* test, respectively.

A propensity score (PS), probability that a patient would have been treated (BICU group), based on all baseline variables, was calculated for each patient [23]. Then, the inverse probability of treatment weighting (IPTW) was calculated as 1/PS for patients in the BICU group, and as 1/(1-PS) for patients in the AICU group [23]. Finally, PS matching (PSM) was conducted to control potential confounding factors and to obtain a balanced retrospective cohort. 1:1 matching using the nearest-neighbor method within a caliper width equal to 0.1 of the SD of the PS was implemented. Standardized mean difference (SMD) was calculated before and after PSM to assess the ‘balance’.

The association between the timing of lactate measures (BICU vs. AICU group) and hospital mortality was analyzed by multivariate logistic regression model for the original cohort (before PSM), IPTW regression model for the original cohort, doubly robust model for the original cohort, and multivariate logistic regression model for the PSM cohort (after PSM). All models were adjusted for covariates that were considered as clinically relevant baseline variables (age, gender, ethnicity, ICU type, admission type, CCI, ICU severity scores, and initial lactate level). The results of the regression models were presented as odds ratio (OR) and 95% confidence intervals (CI).

To clarify mediators affecting the association between the timing of lactate measures (exposure) and hospital mortality (outcome), causal mediation analysis (CMA) was implemented for the PSM cohort. The CMA decomposes the effect of timing of lactate measure on hospital mortality (total effect) into direct effect and indirect effect mediated via mediator [24]. In the present study, therapeutic interventions during the first 24hr after ICU admission were selected as mediator variables.

Additionally, restricted mean survival time (RMST) [21,22] and mean difference of RMSTs between BICU and AICU group were calculated for specific time points (τ): 28- days and 60- days after ICU admission.

Two-sided *p* values <0.05 were considered significant. All analyses were performed using software Stata/SE package version 16.0 (StataCorp, College Station, TX, USA) or the free software package “R” version 4.2.1.

## Results

### Baseline characteristics

**Fig 1:**
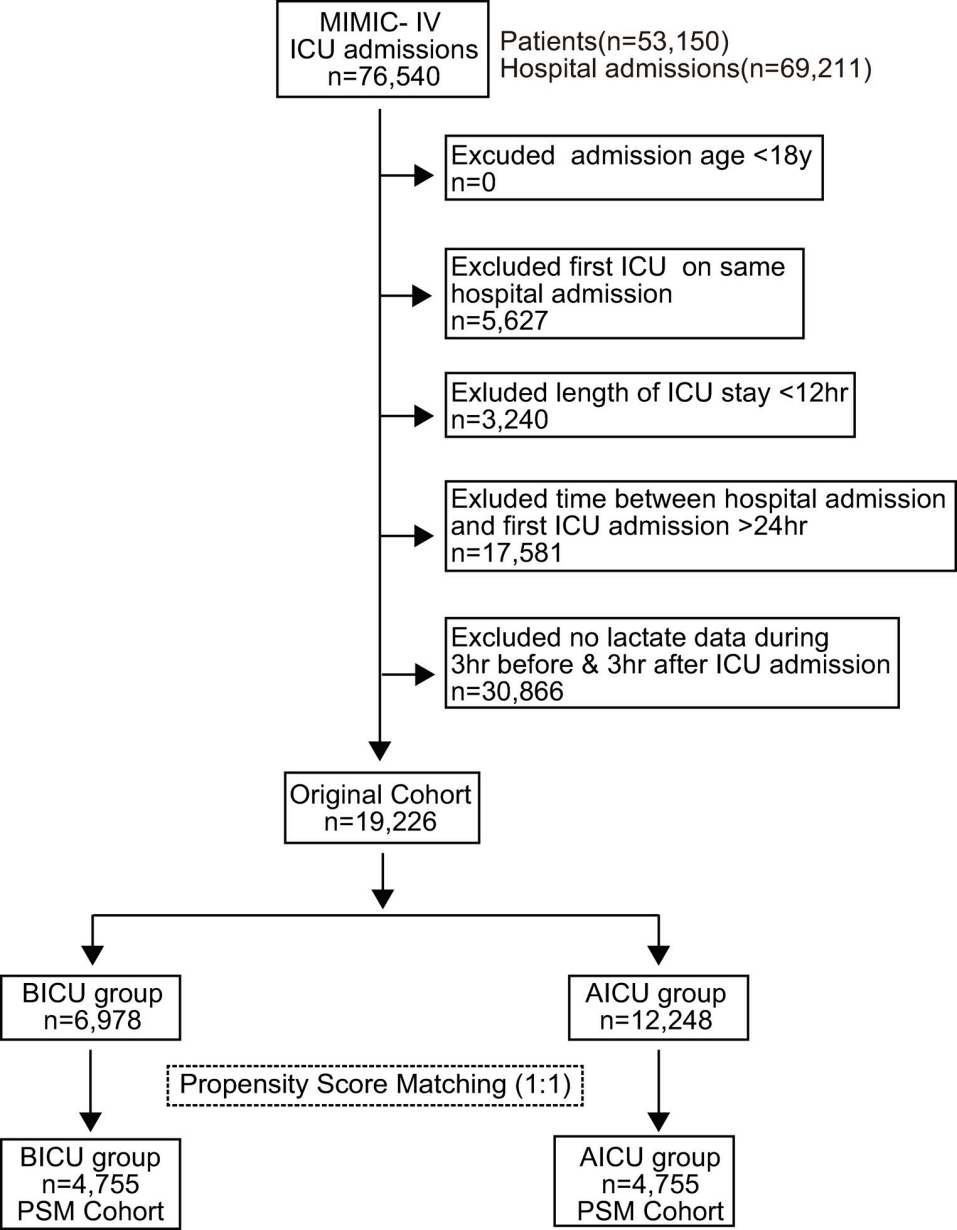
Flow diagram of study patients.

From the MIMIC-Ⅳ database, consisting of 76,540 ICU admissions, 53,150 patients, and 69,211 hospital admission, a total of 19,226 patients were finally included as the original cohort, and divided into BICU group (n = 6,978) and AICU group (n = 12,248) (Fig 1). After 1:1 PSM, 4,755 patients remained in each of the BICU or AICU groups, as the PSM cohort (Fig 1). Comparisons of baseline variables between the two groups in both original and PSM cohorts are summarized in Table 1.

**Table 1:** Comparisons of baseline variable between BICU and AICU groups in original and PSM cohorts.

	Original cohort (n = 19,226)				PSM cohort (n = 9,510)			
Variable	BICU group (n = 6,978)	AICU group (n = 12,248)	SMD	*p* value	BICU group (n = 4,755)	AICU group (n = 4,755)	SMD	*p* value
Age, yr	67 (57-76)	65 (53-77)	0.121	<0.001	65 (55-75)	67 (55-77)	-0.029	0.151
Male gender, *n* (%)	4,343 (62.2)	7,217 (58.9)	0.065	<0.001	2,846 (59.9)	2,858 (60.1)	-0.005	0.786
Ethnicity, *n* (%)				<0.001				0.516
White	5,098 (73.1)	7,656 (62.5)	0.219		3,267(49.6)	3,317 (50.4)	-0.023	
Black	539 (7.7)	1,376 (11.2)	-0.112		455(50.4)	447(49.6)	0.007	
Others	1,341 (19.2)	3,216 (26.3)	-0.165		991(49.0)	1,033(51.0)	0.021	
Admission type, *n* (%)				<0.001				0.805
Observatory & Elective	1,580 (22.7)	1,456(11.9)	0.276		857 (49.4)	877(50.6)	-0.009	
Emergent	4,837 (69.3)	8,008 (65.4)	0.107		3,394(50.0)	3,388(50.0)	0.003	
Urgent	561 (8.0)	2,784 (22.7)	-0.434		504(50.7)	490(49.3)	0.008	
ICU type, *n* (%)				<0.001				0.087
CVICU/CCU	4,089 (58.6)	3,087 (25.2)	0.696		1,978 (49.1)	2,052 (50.9)	-0.052	
TICU/SICU/Neuro surgical ICU	1,551 (22.2)	2,794 (22.8)	0.010		1,480 (51.8)	1,378 (48.2)	0.049	
Medical/Medical Surgical ICU	1,338 (19.2)	6,367 (52.0)	-0.715		1,317 (50.2)	1,305 (49.8)	0.007	
Severity of illness								
SAPS II score	35 (27-43)	37 (28-48)	-0.214	<0.001	35 (27-44)	35 (27-44)	-0.014	0.703
SOFA score (first day)	5 (3-7)	6 (4-9)	-0.361	<0.001	5 (3-7)	5 (3-7)	-0.002	0.942
CCI	5 (3-7)	5 (3-8)	-0.085	<0.001	5 (3-7)	5 (3-7)	-0.008	0.607
Sepsis-3,(first day) *n* (%)	4,262 (61.1)	8,268 (67.5)	-0.161	<0.001	2,797 (58.2)	2,779 (58.4)	-0.009	0.708
Initial lactate level (mmol/L)	1.6 (1.1-2.3)	1.8 (1.3-2.9)	-0.189	<0.001	1.7 (1.2-2.6)	1.8 (1.2-2.7)	-0.018	0.318
Interventions (first day)								
Mechanical ventilation, *n* (%)	3,490 (50.0)	6,055 (49.4)	-0.016	0.441	2,181 (45.9)	2,173 (45.7)	0.006	0.869
Vasopressors, *n* (%)	1,465 (21.0)	4,058 (33.1)	-0.343	<0.001	1,032 (21.7)	1,011 (21.3)	0.007	0.600
RRT, *n* (%)	175 (2.5)	765 (6.3)	-0.185	<0.001	156 (3.3)	159 (3.3)	-0.003	0.864
Vital Signs(first day)								
Heart rate (bpm)	82 (75-92)	86 (76-99)	-0.255	<0.001	84 (76-95)	84 (75-95)	0.022	0.283
MAP (mmHg)	75 (71-81)	76 (70-83)	-0.095	<0.001	76 (71-83)	76 (70-82)	0.039	0.178
Respiratory rate (/min)	18 (16-20)	19 (17-22)	-0.409	<0.001	18 (16-21)	18 (16-21)	-0.015	0.292
Temperature (C°)	36.8 (36.5-37.0)	36.8 (36.6-37.2)	-0.127	<0.001	36.8 (36.6-37.1)	36.8 (36.6-37.1)	0.029	0.960
SpO_2_(%)	98 (96-99)	97 (96-99)	0.211	<0.001	98 (96-99)	98 (96-99)	0.022	0.798
Laboratory data (first day)								
Hemoglobin (g/dL)	10.3 (9.3-11.5)	10.6 (9.2-12.2)	-0.100	<0.001	10.5 (9.4-11.8)	10.5 (9.2-11.9)	0.012	0.477
Hematocrit (%)	31 (28-34)	32 (28-37)	-0.178	<0.001	31.6 (28.4-35.3)	31.6 (28.0-35.7)	0.009	0.565
WBC (×10^3^/mm^3^)	12.0 (9.1-15.4)	11.8 (8.5-15.9)	-0.065	<0.001	11.8 (8.8-15.4)	11.4 (8.4-15.0)	0.017	0.380
Platelet (×10^3^/μL)	161 (125-212)	187 (134-254)	-0.227	<0.001	174 (131-233)	177 (131-235)	0.008	0.291
Sodium (mEq/L)	139 (137-140)	139 (136-141)	-0.030	<0.05	139 (136-141)	139 (136-141)	0.006	0.466
Chloride (mEq/L)	107 (104-109)	105 (100-108)	0.292	<0.001	107 (103-108)	106 (102-109)	0.026	0.183
Potassium (mEq/L)	4.3 (4.0-4.6)	4.2 (3.8-4.6)	0.013	<0.001	4.2 (3.9-4.6)	4.2 (3.9-4.6)	-0.005	0.378

SMD: Standardized mean difference, CVICU: Cardiovascular ICU, CCU: Coronary care unit, TICU: Trauma ICU, SICU: Surgical ICU, CCI: Charlson comorbidity index, RPT: Renal replacement therapy. All values are expressed as number (n) (%) or median(IQR). All values of vital signs and laboratory data are averaged.

In the PSM cohort, SMD for all baseline variables was ≤ 0.051, indicating similar distribution, and there were no significant differences in variables between the two groups.

Initial lactate level, minutes to measure lactate, and total number of measured lactates during each time-window in the PSM cohort are summarized in S1 Table. Patients included in -3 hr to -2 hr, -2 hr to -1 hr, and -1 hr - 0 hr before ICU (BICU group) were 1,785, 1,724, and 1,246, respectively (4,755 in total). Similarly, patients included in 0 hr -1 hr, 1 hr- 2 hr, and 2 hr - 3 hr after ICU admission (AICU group) were 2,027, 1,672, and 1,056, respectively (4,755 in total) (S1 Table).

Comparisons of baseline variables between survivors and non-survivors in total, BICU, and AICU groups in the PSM cohort are summarized in S2 Table.

### Primary outcome

In both original (n = 19,226) and PSM (n = 9,510) cohorts, hospital mortality rate differed significantly between the two groups (5.4% for BICU and 16.0% for AICU group in the original cohort, and 7.0% for BICU and 9.8% for AICU group in the PSM cohort, *p*<0.001 for both) (Table 2).

**Table 2:** Primary and secondary outcomes in original and PSM cohort.

Outcomes	BICU group	AICU group	Difference	*p* value
PSM cohort (n = 9,510)	(n = 4,755)	(n = 4,755)		
Hospital mortality (%)	7.0 (6.3-7.8)	9.8 (9.0-10.7)	2.8 (1.7-3.9)	<0.001
Hospital 28day mortality (%)	6.7 (6.0-7.4)	9.4 (8.5-10.2)	2.7 (1.6-3.8)	<0.001
ICU mortality (%)	4.6 (4.0-5.2)	6.7 (6.0-7.4)	2.1 (1.2-3.1)	<0.001
LOS hospital (days)	8.4 (8.2-8.7)	9.0 (8.7-9.3)	0.6 (0.2-0.9)	<0.001
LOS ICU (days)	3.0 (2.9-3.1)	3.5 (3.4-3.6)	0.5 (0.4-0.7)	<0.001
RMST for 28-day	24.7 (24.3-25.0)	24.1 (23.8-24.5)	0.531 (0.002-1.059)	<0.05
RMST for 60-day	46.9(45.1-48.8)	45.7(44.0-47.3)	1.260(-1.233-3.754)	0.322
Original cohort (n = 19,226)	(n = 6,978)	(n = 12,248)		
Hospital mortality (%)	5.4 (4.9-6.0)	16.0 (15.4-16.7)	10.6 (9.8-11.5)	<0.001
Hospital 28day mortality (%)	5.1 (4.6-5.7)	15.3 (14.7-16.0)	10.2 (9.4-11.0)	<0.001
ICU mortality (%)	3.7 (3.2-4.1)	11.7 (11.1-12.3)	8.0 (7.3-8.8)	<0.001
LOS hospital (days)	8.0 (7.8-8.2)	9.9 (9.7-10.1)	1.9 (1.6-2.2)	<0.001
LOS ICU (days)	2.9 (2.8-3.0)	4.3 (4.2-4.4)	1.3 (1.2-1.5)	<0.001
RMST for 28-day	25.1 (24.8-25.5)	22.6 (22.4-22.9)	2.506 (2.112-2.901)	<0.001
RMST for 60-day	48.2 (46.5-49.9)	42.2 (41.3-43.1)	6.034 (4.146-7.922)	<0.001

LOS: Length of stay, RMST: Restricted mean survival time. All values are expressed as mean (95%CI).

The results of the 4 multivariate regression models for hospital mortality after adjustment for the predefined covariates are shown in Table 3.

**Table 3:** Multivariate regression model for hospital mortality.

Model	OR* (95%CI)	*Z* value	*p* value
Multivariate regression model for original cohort	0.50 (0.44-0.57)	-10.08	<0.001
IPTW logistic regression model for original cohort	0.96 (0.95-0.97)	-5.18	<0.001
Doubly Robust regression model for original cohort	0.52 (0.46-0.60)	-9.36	<0.001
Multivariate regression model for PSM cohort	0.66 (0.56-0.78)	-4.86	<0.001

*Adjusted for gender, age, ethnicity, ICU type, admission type, CCI, ICU severity scores, and initial lactate level. IPTW: Inverse probability treatment weight.

There was a significant OR for hospital mortality in all 4 models (range of ORs: 0.50-0.96, *p*<0.001 for all). Thus, lactate measures within 3 hr prior to ICU admission was suggested as a risk-adjusted factor for lower hospital mortality.

### Secondary outcomes

Results of secondary outcomes in both cohorts are shown in Table 2.

**Fig 2:**
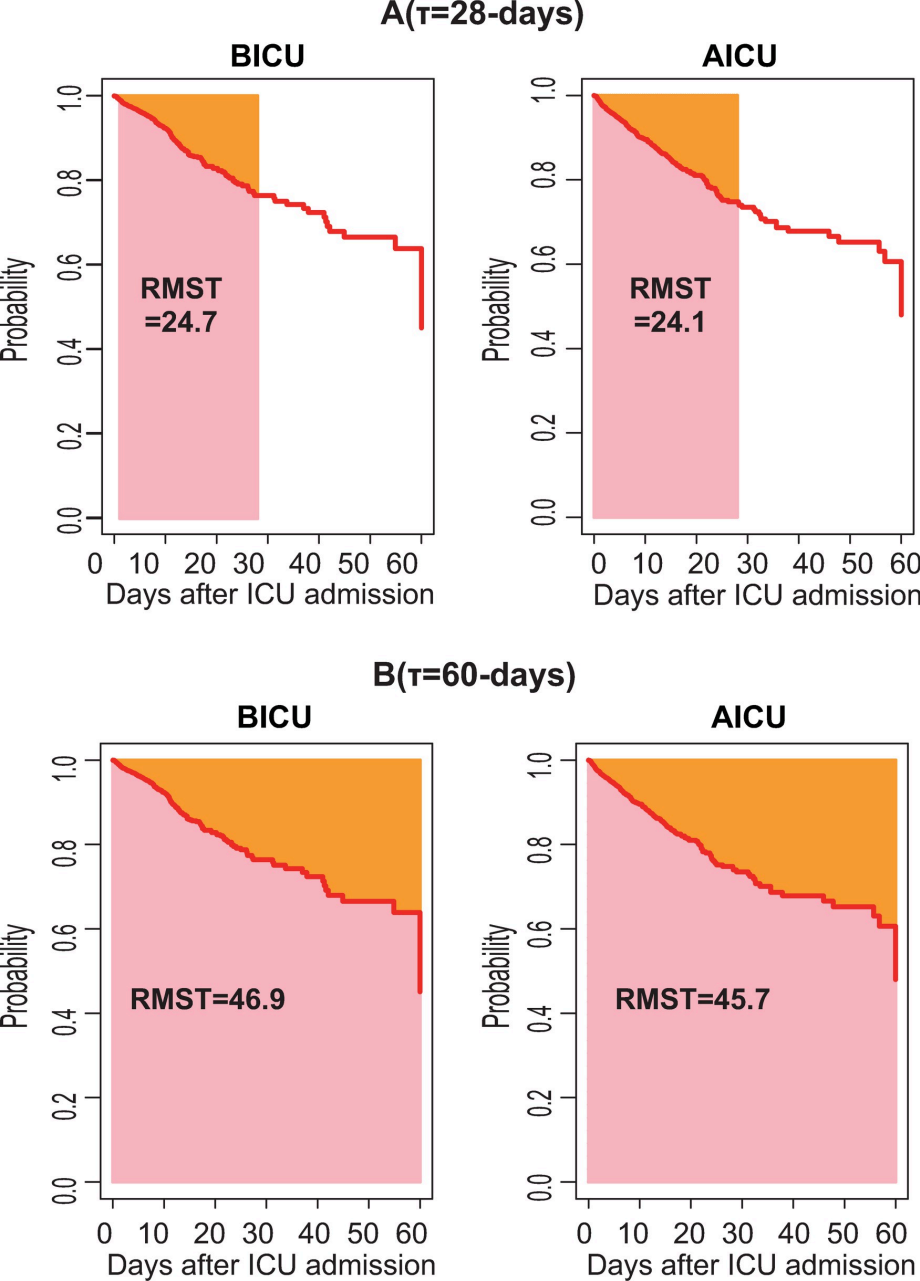
Comparisons of restricted mean survival time (RMST) for τ = 28-days(A) and τ = 60-days(B) between the two group in the PSM cohort (n = 9,510).

In the PSM cohort, both hospital 28-day mortality rate and ICU mortality rate differed significantly between the two groups (6.7% for BICU vs.9.4% for AICU group, and 4.6% for BICU vs. 6.7% for AICU group, respectively, *p*<0.001 for both). Hospital LOS and ICU LOS were also different between the two groups (8.4 days for BICU vs. 9.0 days for AICU group, and 3.0 days for BICU vs. 3.5 days for AICU group, respectively, *p*<0.001 for both). RMST at 28-days after ICU admission was significantly different between the two groups (24.7 days for BICU vs. 24.1 days for AICU group, a difference of 0.531 days, [95%CI, 0.002- 1.059], *p*<0.05), but not for RMST at 60-days (46.9 days for BICU vs. 45.7 days for AICU group, a difference of 1.260 days, [95%CI, -1.233–3.754], *p* = 0.322) (Fig 2).

In the original cohort, there were highly statistically significant differences in hospital mortality, hospital 28-day mortality, ICU mortality, hospital LOS, ICU LOS, and both the RMSTs at 25-days and 60-days between the two groups (*p*<0.001 for all) (S1 Fig).

### Subgroup analysis

**Fig 3:**
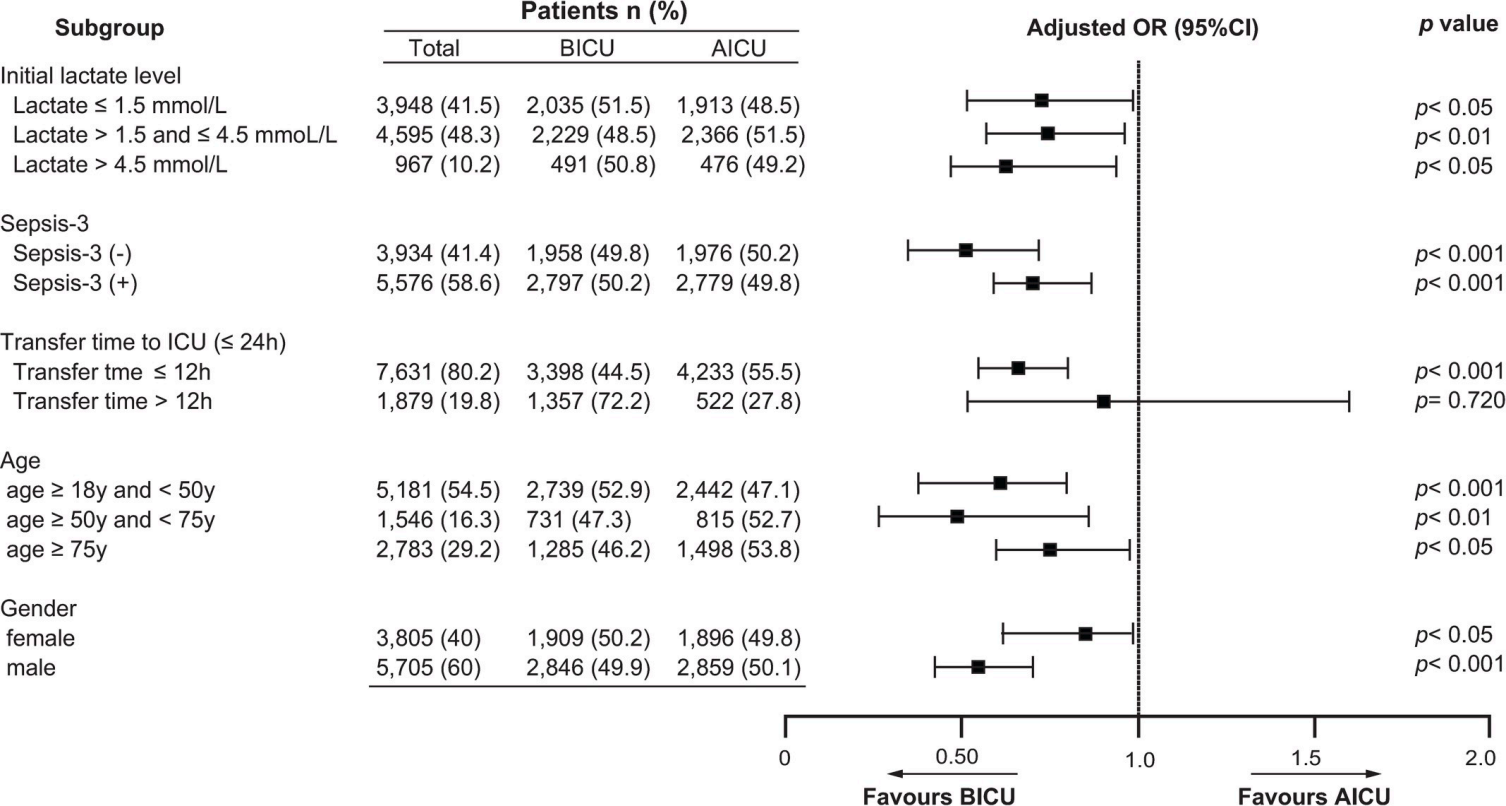
Subgroup analysis of adjusted OR for hospital mortality in the PSM cohort (n = 9,510). OR was adjusted for age, gender, ethnicity, ICU type, admission type, ICU severity scores, CCI, and initial lactate level. Error bars represent 95% confidence intervals.

**Fig 4:**
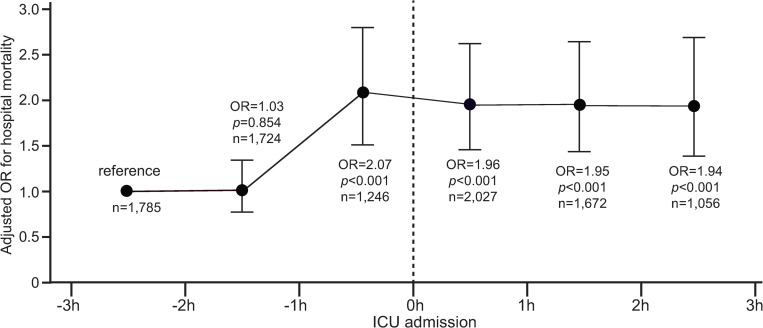
Subgroup analysis of adjusted OR for hospital mortality during each time-window for lactate measures in the PSM cohort (n = 9,510). OR was adjusted for age, gender, ethnicity, ICU type, admission type, ICU severity scores, CCI, and initial lactate level. Error bars represent 95% confidence intervals.

Two subgroup analyses were implemented in the PSM cohort and results are shown in Figs 3 and 4, respectively.

Results of the subgroup analysis of initial lactate level, first- day Sepsis-3, transfer time, age, and gender are shown in Fig 3. For initial lactate level (lactate ≤1.5 mmol/L,1.5 < lactate ≤ 4.5, lactate > 4.5), adjusted ORs for hospital mortality were 0.71 (95%CI,0.52-0.98), *p*<0.05, 0.715 (95% CI, 0.56-0.91), *p*<0.01, and 0.66 (95%CI, 0.46-0.94), *p*<0.05, respectively. Similarly, For Sepsis-3 (yes/no), adjusted ORs were 0.70 (95%CI, 0.58-0.85), *p*<0.001, and 0.50 (95%CI, 0.35-0.71), *p*<0.001,respectively. For age (age < 50 y, 50 y ≤ age < 75 y, age ≥ 75 y), adjusted ORs were 0.63 (95%CI, 0.49-0.81), *p*<0.001, 0.46 (95%CI, 0.26-0.82), *p*<0.01, and 0.75 (95%CI, 0.59-0.95), *p*<0.05, respectively. For gender (female/male), adjusted ORs were 0.82 (95%CI, 0.65-0.98), *p*<0.05, and 0.55 (95%CI, 0.44-0.69),*p*<0.001, respectively.

However, for transfer time to ICU (≤ 12hr and >12hr), adjusted ORs were 0.65 (95%CI, 0.54-0.79), *p*<0.001, and 0.84 (95%CI, 0.49-1.48), *p* = 0.573, respectively. Thus, the transfer time was suggested as a decisive factor for lower hospital mortality.

Results of subgroup analysis of adjusted ORs for hospital mortality during each time-window for lactate measures are shown in Fig 4. Compared to the reference OR of 1.0 during -3 hr to -2 hr before ICU, adjusted ORs during -1 hr- 0 hr before ICU, during 0 h-1 hr, 1 hr-2 hr, and 2 hr-3 hr after ICU admission were significantly larger (2.07 [95%CI, 1.53 -2.79], 1.96 [95%CI, 1.49-2.59], 1.95 [95%CI,1.47-2.60], 1.94 [95%CI,1.40-2.69], respectively, *p*<0.001 for all).

### Interventions within the first 24hr after admission to ICU

Comparisons of the first 24 hr therapeutic interventions between the two groups in the PSM cohort are summarized in Table 4.

**Table 4:** Comparisons of therapeutic intervention during first 24h after ICU admission in the PSM cohort.

Therapeutic Interventions	BICU group (n = 4,755)	AICU group (n = 4,755)	*p* value
Number of lactate measures			
During first 3hr	6,986	6,167	<0.001
During first 6hr	10,359	8,406	<0.001
Mechanical ventilation			
Number (%)	2,181 (45.9)	2,173 (45.7)	0.869
Minutes to Ventilation	116 (28-208)	101 (13-272)	0.617
Vasopressors			
Number (%)	1,032 (21.7)	1,011 (21.3)	0.600
Minutes to vasopressors	173 (80-282)	66 (17-264)	<0.001
Number of vasopressors	1 (1-2)	1 (1-2)	0.743
RRT			
Number (%)	156 (3.3)	159 (3.3)	0.864
Minutes to RRT	288 (103-835)	233 (77-721)	0.342
A-line			
Number (%)	3,220 (67.7)	2,440 (51.3)	<0.001
Minutes to A-line	126 (44-217)	197 (85-337)	<0.001
CV-line			
Number (%)	1,783 (37.5)	1,740 (36.6)	0.361
Minutes to CV-line	160 (81-266)	159 (39-344)	0.178
PA-catheter			
Number (%)	930 (19.6)	626 (13.2)	<0.001
Minutes to PA-catheter	182 (109-306)	274 (132-367)	<0.001
Administration of IV antibiotics			
Number (%)	3,686 (77.5)	3,214 (67.6)	<0.001
Minutes to IV antibiotics	109 (49-253)	168 (85-327)	<0.001
Bolus fluid infusion			
Number (%)	2,738 (57.6)	2,455 (51.6)	<0.001
Minutes to bolus	224 (137-354)	278 (116-450)	<0.001

RRT: Renal replacement therapy; A-line: Arterial line; CV-line: Central venous line; PA-catheter: Pulmonary arterial catheter. Mechanical ventilation includes non- invasive ventilation. Vasopressors include norepinephrine, epinephrine, vasopressin, dobutamine, dopamine, and phenylephrine. All values are expressed as number (%) or median (IQR).

There was a significant difference in total number of lactate measures during the first 3 hr (6,986 for BICU vs. 6,167 for AICU group, *p*<0.001) and during the first 6 hr (10,359 for BICU vs.8,406 for AICU group, *p*<0.001).

Furthermore, placement of A-line and PA-catheter, and administration of IV antibiotics were significantly more frequent and faster in the BICU group (A-line: 67.7% vs. 51.3% and 126 min vs. 197 min, respectively, *p*<0.001 for both, PA-catheter: 19.6% vs.13.2% and 182 min vs. 274 min, respectively, *p*<0.001 for both, IV antibiotics: 77.5% vs. 67.6% and 109 min vs. 168 min, respectively, *p*<0.001 for both). Additionally, IV bolus fluid infusion in the BICU group was administered significantly more frequently and faster (57.6% vs. 51.6% and 224 min vs. 278 min, respectively, *p*<0.001 for both).

However, there were no significant differences in the use of mechanical ventilation, use of vasopressors, use of RRT, and placement of CV-line between the two groups. To the contrary, minutes to vasopressors was significantly shorter in the AICU group (173 min vs. 66 min, p<0.001).

### Causal mediation analysis

The CMA allows decomposing the total effect of an exposure on an outcome into a direct effect of the exposure on the outcome and an indirect effect that acts through a mediator of interest by using the counterfactual approach [7,11, 24]. Several methods and software programs have been developed for CMA [25]. We adopted the difference method based on fitting two parametric regression models [26]. The results of the CMA for therapeutic intervention are summarized in S3 Table. There were significant indirect effects in frequency and time of A-line replacement (0.19879 [95%CI, 0.14061-0.25697], p<0.001 and 0.07714 [95%CI, 0.22600-0.13168], p<0.01,respectively). Additionally, significant indirect effects were also observed in frequency of PA-catheter replacement and bolus fluid infusion (0.05614 [95%CI, 0.04088-0.07140], p<0.001, and 0.02193 [95% CI, 0.00303-0.04083], p<0.05,respectively). Thus, the beneficial effects of lactate measures before ICU admission are suggested to be partly mediated through the three therapeutic interventions.

## Discussion

We performed a simple comparative study of measuring lactates before vs. after ICU admission in unselected heterogeneous ICU patients extracted from the MIMIC-IV database. Consequently, in comparison with measures within 3 hr after ICU admission, lactate measures within 3 hr prior to ICU admission were significantly associated with lower risk-adjusted hospital mortality in 4 different multivariate regression models (Table 3).

In particular, the IPTW model generates a new weighted score for treatment and control on individual patients, preventing the loss of PS unmatched patients in the PSM cohort, and mimics a randomized controlled trial by avoiding selection bias [23,27]. Additionally, the doubly robust model requires one model for the outcome and another model for the exposure but is consistent if either model is correct, not necessary both, providing double chances to make a valid inference [27,28]. Both IPTW and doubly robust models are generally accepted to efficiently evaluate average treatment effect (ATE) [27,28].

### Timing of measuring lactates

It seems rational that the latest lactate data prior to ICU may trigger prompt responses and enable rapid therapeutic interventions.

For the septic patients, the surviving sepsis campaign recommends “Hour-1 Bundle”: when initial lactate level > 2 mmol/L, it should be remeasured within 2–4 hr to guide resuscitation to normalize lactate in patients with elevated lactate levels as a marker of tissue hypoperfusion [29]. Chen et al. [7] have clearly documented that early lactate measurement within 1hr after ICU admission was significantly associated with lower 28-day mortality, probably, due to a shorter time to vasopressors in septic patients with an initial lactate level >2.0 mmol/L extracted from the MIMIC-III database, and that the adequate time interval between early and the second measurement was within 3 hr. Similarly, Chen et al. [11] have shown that early lactate measurement within 1 hr after ICU admission was significantly associated with lower 28-day mortality, mediated through shorting the time to initial IV fluid, in adult patients with hypotension and hyperlactatemia extracted the MIMIC-III and eICU Collaborative Research Database. Unfortunately, no lactate data prior to ICU admission were shown in either of those studies.

In contrast, our study investigated the association of the timing of measuring lactate relevant to ICU admission and hospital mortality in unselected heterogeneous critically ill patients with normal to elevated blood lactate levels.

### Subgroup analysis

In the subgroup analysis of transfer time, the patients with delayed transfer time to ICU (> 12 hr) did not show a significant OR for hospital mortality (Fig 3). Several other studies [30–32] have indicated that delayed transfer time to ICU was associated with poor outcomes. Churpek et al. [31] described a significant association of delayed transfer time (≥ 6hr) and increased hospital mortality in 3,789 medical-surgical ward patients. Similarly, Chalfin et al.[32] described that ED critically ill patients with ≥ 6hr delay to ICU (n = 1,036) had increased hospital LOS, higher rate to admit higher level of ICU, and higher hospital mortality in 50,322 ICU patients. Considering the transfer time from hospital admission to ICU admission in this study, not from ward or ED, it appears to be comparable to time delay of 6 hr in the Chalfin or Chalfin study.

In the subgroup analysis of 3 lactate levels, even the patients with normal lactate levels ≤ 1.5mmol/L showed a significant OR for hospital mortality (Fig 3). A normal lactate level in unstressed individuals is 1.0±0.5 mmol/L [33]. However, Nicol et al.[33] documented significantly higher ORs for hospital mortality at lactate levels of 0.75-1.0,1.01-1.25, and 1.26-1.50 mmol/L on ICU admission, compared to OR of 1.0 at lactate levels of 0-0.75 mmol/L in 7,155 ICU patients. Additionally, Chebl et al.[17] documented that critically ill patients with normal lactate levels on ICU admission (49.6%) had a high hospital mortality rate of 19.6% and ICU mortality rate of 9.8% in 450 ED patients.

### Restricted mean survival time (RMST)

RMST estimates are the truncated averaged area from the start of follow-up to a predefined follow-up time point (τ) under the Kaplan-Meier survival curve [21,22]. In other words, RMST means the τ-specific life expectancy [22]. The specific points (τ) of 28-days and 60-days in this study were chosen based on a study by Zhou et al. [8], who evaluated the timing of albumin administration in septic patients extracted from the MIMIC-IV database.

RMST difference between treatment and control groups means an interpretable and intuitive expression of ATE, but with a relatively small effect size [34].

Actually, in the PSM cohort, the RMST difference for 28- days (τ) was small (0.53 days, *p*<0.05), partially explained by the concomitant small difference of ICU LOS (0.5 days) or hospital LOS (0.6 days) (Table 2, Fig 2).

### Limitations

The present study has several limitations. First, this retrospective study was based on 7 ICUs at a single institutional database. Thus, the treatments against elevated lactate levels were not protocoled or uniformly reported due to the retrospective nature of the study and unselected heterogeneous cohort. Second, there were no statistically significant differences between the two groups in categorical data (ethnicity, admission type, and ICU type) after PSM. However, the individual classification for the categorical data was arbitrarily assigned, not based on explicit reasons, may leading to selection bias. Third, the measured lactate in both groups was not always the first measure after hospital admission. Therefore, therapeutic interventions after the first measure were not considered. Finally, unpredictable confounders and selection biases might exist, affecting the present findings. Thus, further prospective randomized trials are needed.

## Conclusions

In unselected heterogeneous ICU patients with transfer time to ICU admission (≤ 24hr), measuring lactate within 3 hr prior to ICU might be associated with lower hospital mortality presumably due to more frequent and faster therapeutic interventions.

## Supporting information

S1 Fig Comparisons of restricted mean survival time (RMST) for τ = 28-days (A) and τ = 60-days (B) between the two groups in the original cohort (n = 19,226).
(TIF)

S1 Table Initial lactate level (mmol/L), minutes to measure lactate, and total number of lactate measures (n) during each time interval in the PSM cohort. Values are expressed as median value of initial lactate level (IQR), median value of minutes to measure lactates (IQR), and total number of lactate measures (n).
(DOCX)

S2 Table Comparisons of baseline variables between survivors and non-survivors in BICU and AICU groups in the PSM cohort. CVICU: Cardiovascular ICU, CCU: Coronary care unit, TICU: Trauma ICU, SICU: Surgical ICU, CCI: Charlson comorbidity index, RPT: Renal replacement therapy. All values are expressed as n (%) or median (IQR). All values of vital signs and laboratory data are averaged.
(DOCX)

S3 Table Causal mediation analysis for therapeutic intervention in the PSM cohort. Direct and indirect effects are derived from when age, CCI, ICU severity scores, and initial lactate level are fixed at the mean values. Mechanical ventilation includes non-invasive ventilation. Vasopressors include norepinephrine, epinephrine, vasopressin, dobutamine, dopamine, and phenylephrine.
(DOCX)

## Abbreviations

ATE: average treatment effect
CI: Charlson comorbidity index
CMA: causal mediation analysis
CV: central venous
CI: confidence interval
ED: emergency department
ICU: intensive care unit
IPTW: inverse probability of treatment weighting
IQR: interquartile range
LOS: length of stay
MIMIC: medical information mart for intensive care
OR: odds ratio
PS: propensity score
PSM: propensity score matching
PA: pulmonary artery
RMST: restricted mean survival time
RRT: renal replacement therapy
SAPS: severity acute physiologic score
SD: standard deviation
SMD: standardized mean difference
SQL: structured query language
SOFA: sequential organ failure assessment
