# Supplementary figures and images for: Impact on outcomes of measuring lactates prior to ICU in unselected heterogeneous critically ill patients: A propensity score analysis
Source: PLoS One. 2022 Nov 28;17(11):e0277948. doi: 10.1371/journal.pone.0277948 (PMC9704607; doi:10.1371/journal.pone.0277948)

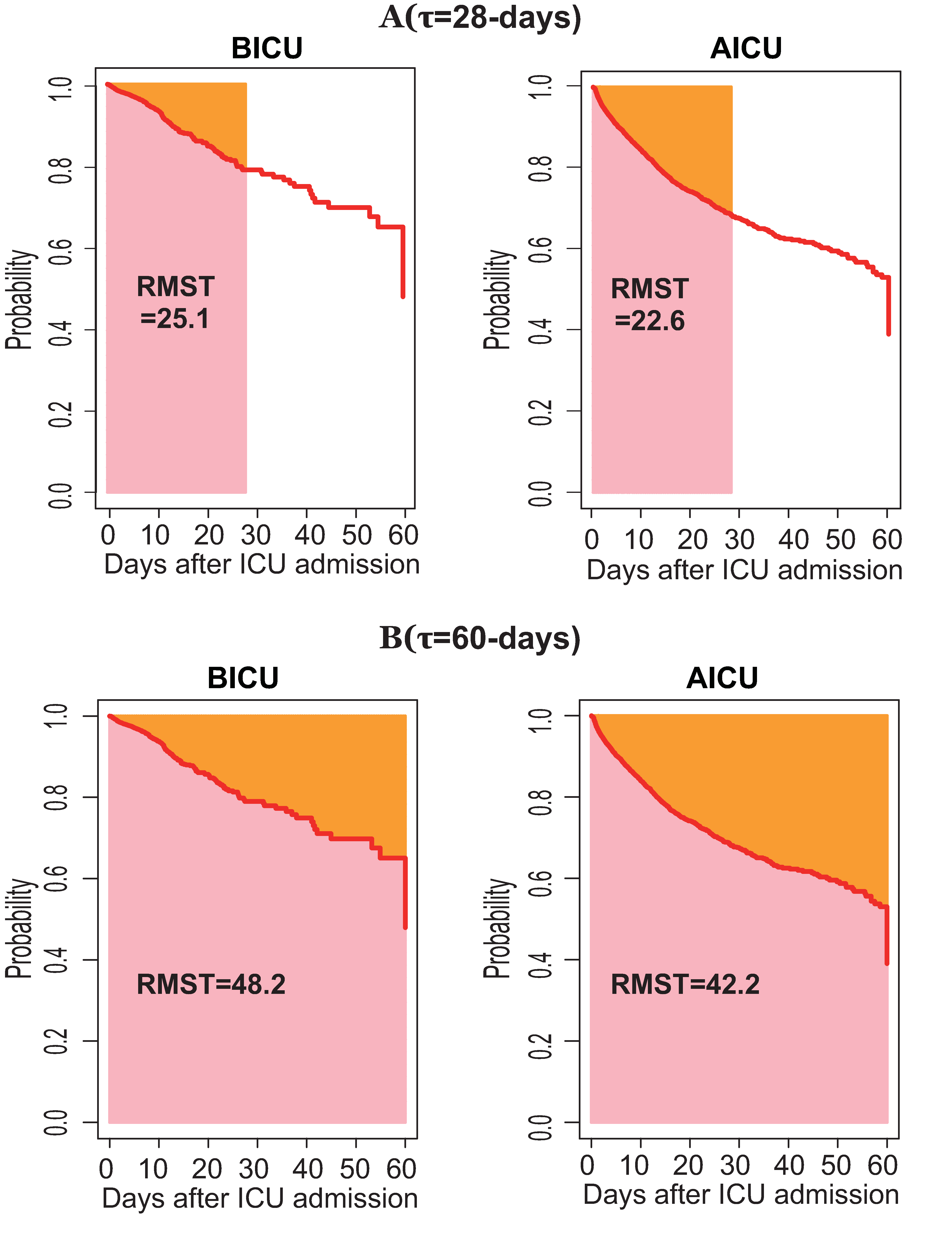

Supplement: S1 Fig — Comparisons of restricted mean survival time (RMST) for τ = 28-days (A) and τ = 60-days (B) between the two groups in the original cohort (n = 19,226). (TIF) [file pone.0277948.s001.tif]
