# Supplementary material for: Impact on outcomes of measuring lactates prior to ICU in unselected heterogeneous critically ill patients: A propensity score analysis
Source: PLoS One. 2022 Nov 28;17(11):e0277948. doi: 10.1371/journal.pone.0277948 (PMC9704607; doi:10.1371/journal.pone.0277948)
Supplement: S1 Table — Values are expressed as median value of initial lactate level (IQR), median value of minutes to measure lactates (IQR), and total number of lactate measures (n). (DOCX) [file pone.0277948.s002.docx]

**Table S1.** **Initial lactate level (mmol/L), minutes to measure lactate, and total number of measured lactates (n) during each time-window in the PSM cohort**

|  |  | **Before ICU admission** | | | **After ICU admission** | | | | | | | | |  |
| --- | --- | --- | --- | --- | --- | --- | --- | --- | --- | --- | --- | --- | --- | --- |
|  |  | -3h to -2h | -2h to -1h | -1h- 0h | 0h-1h | 1h-2h | 2h-3h | 3h-4h | 4h-5h | 5h-6h | 6h-7h | 7h-8h | 8h-9h |  |
| BICU  group  (n=4,755**)** | Patients n=1,785 | 1.8  mmol/L  (1.2-2.7)  -148 min  (-163 to-134）  n=1,840 | 2.7  mmol/L  (1.8-4.4)  -88 min  (-101 to -72)  n=406 | 2.5  mmol/L  (1.8-3.7)  -34 min  (-48 to -20)  n=455 | 2.3  mmol/L  (1.8-3.2)  30 min  (17-44)  n=625 | 2.4 mmol/L  (1.7-3.3)  87 min  (72-101)  n=465 | 2.6 mmol/L  (1.8-3.7)  146 min  (131-161)  n=271 | 2.4 mmol/L  (1.6-3.4)  208 min  (192-223)  n=235 | 2.5 mmol/L  (1.7-3.9)  269 min  (254-283)  n=232 | 2.4 mmol/L  (1.7-3.7)  327 min  (312-343)  n=196 | 2.6 mmol/L  (1.5-3.8)  391 min  (373-406)  n=180 | 2.3 mmol/L  (1.7-3.4)  455 min  (438-468)  n=174 | 2.4 mmol/L  (1.5-3.6)  505 min  (495-519)  n=182 |  |
|  |  | Patients  n=1,724 | 1.6  mmol/L  (1.2-2.4)  -91 min  (-106 to -75)  n=1,763 | 2.5 mmol/L  (1.7-3.4)  -29 min  (-44 to -15)  n=284 | 2.2  mmol/L  (1.6-3.0)  30 min  (19-44)  n=487 | 2.3 mmol/L  (1.7-3.0)  87 min  (74-101)  n=575 | 2.3 mmol/L  (1.8-3.2)  147 min  (134-162)  n=403 | 2.5 mmol/L  (1.6-3.8)  206 min  (194-224)  n=290 | 2.4 mmol/L  (1.7-3.6)  269 min  (255-285)  n=230 | 2.2 mmol/L  (1.6-3.3)  330 min  (312-342)  n=185 | 2.4 mmol/L  (1.6-3.7)  389 min  (376-406)  n=182 | 2.2 mmol/L  (1.5-3.6)  449 min  (434-461)  n=166 | 2.5 mmol/L  (1.7-3.7)  510 min  (497-525)  n=157 |  |
|  |  |  | Patients  n=1,246 | 1.7 mmol/L  (1.2-2.7)  -31 min  (-46 to-16)  n=1,281 | 2.2 mmol/L  (1.5-3.6)  35 min  (20-46)  n=190 | 2.2 mmol/L  (1.5-3.6)  91 min  (77-105)  n=280 | 2.2 mmol/L  (1.6-3.2)  149 min  (132-164)  n=308 | 2.4 mmol/L  (1.7-3.5)  208 min  (194-220)  n=263 | 2.9 mmol/L  (1.6-4.4)  267 min  (254-281)  n=173 | 2.6 mmol/L  (1.7-4.4)  332 min  (314-344)  n=155 | 2.5 mmol/L  (1.5-3.9)  387 min  (375-403)  n=153 | 2.1 mmol/L  (1.4-3.8)  450 min  (436-465)  n=140 | 2.1 mmol/L  (1.5-4.5)  512 min  (497-526)  n=133 | |
|  |  |  | AICU  group  (n=4,755) | Patients  n=2,027 | 1.8 mmol/L  (1.3-2.9)  34 min  (20-47)  n=2,109 | 2.8 mmol/L  (1.8-4.6)  94 min  (77-107)  n=272 | 2.4 mmol/L  (1.7-3.8)  150 min  (137-166)  n=393 | 2.3 mmol/L  (1.6-3.5)  209 min  (195-224)  n=428 | 2.4 mmol/L  (1.7-3.6)  267 min  (253-284)  n=395 | 2.5 mmol/L  (1.6-4.2)  329 min  (314-345)  n=304 | 2.2 mmol/L  (1.5-3.8)  390 min  (374-404)  n=274 | 2.3 mmol/L  (1.4-4.0)  450 min  (437-464)  n=247 | 2.3 mmol/L  (1.5-3.7)  508 min  (496-525)  n=239 |  |
|  |  |  |  |  | Patients  n=1,672 | 1.8 mmol/L  (1.2-2.6)  85 min  (72-100)  n=1,718 | 2.2 mmol/L  (1.6-3.6)  154 min  (140-167)  n=154 | 2.2 mmol/L  (1.6-3.3)  210 min  (195-227)  n=253 | 2.2 mmol/L  (1.7-3.2)  269 min  (253-282)  n=297 | 2.2 mmol/L  (1.5-3.2)  327 min  (312-345)  n=242 | 2.2 mmol/L  (1.5-3.5)  388 min  (375-403)  n=207 | 1.9 mmol/L  (1.3-3.0)  448 min  (434-463)  n=196 | 1.9 mmol/L  (1.4-3.3)  512 min  (496-527)  n=170 |  |
|  |  |  |  |  |  | Patients  n=1,056 | 1.7 mmol/L  (1.2-2.5)  145 min  (131-162)  n=1,076 | 2.4 mmol/L  (1.5-3.8)  216 min  (203-229)  n=78 | 2.2 mmol/L  (1.6-3.0)  270 min  (257-284)  n=114 | 2.0 mmol/L  (1.5-2.8)  329 min  (314-341)  n=126 | 2.2 mmol/L  (1.5-3.0)  386 min  (374-401)  n=130 | 1.9 mmol/L  (1.5-2.8)  453 min  (437-463)  n=110 | 1.8 mmol/L  (1.3-2.8)  512 min  (499-525)  n=95 |  |

All Value are expressed as median of initial lactate level (IQR), median of min to measure lactates (IQR), and total number of lactate measures (n).
