# Supplementary material for: Impact on outcomes of measuring lactates prior to ICU in unselected heterogeneous critically ill patients: A propensity score analysis
Source: PLoS One. 2022 Nov 28;17(11):e0277948. doi: 10.1371/journal.pone.0277948 (PMC9704607; doi:10.1371/journal.pone.0277948)
Supplement: S2 Table — CVICU: Cardiovascular ICU, CCU: Coronary care unit, TICU: Trauma ICU, SICU: Surgical ICU, CCI: Charlson comorbidity index, RPT: Renal replacement therapy. All values are expressed as n (%) or median (IQR). All values of vital signs and laboratory data are averaged. (DOCX) [file pone.0277948.s003.docx]

**Table S2.** **Comparisons of baseline variables between survivors and non-survivors in BICU and AICU groups in the PSM cohort**

|  | Total（n=9,510） | | | BICU group(n=4,755) | | | AICU group(n=4,755) | | |
| --- | --- | --- | --- | --- | --- | --- | --- | --- | --- |
| Variable | Survivors  (n=8,708) | Non-survivors  (n=802) | *p* value | Survivors  (n=4,420) | Non-survivors  (n=335) | *p* value | Survivors  (n=4,288) | Non-survivors  (n=467) | *p* value |
| Age, yr | 65 (54-75) | 74 (62-83) | <0.001 | 65 (55-75) | 73 (60-82) | <0.001 | 66 (54-76) | 75 (62-84) | <0.001 |
| Male gender, *n* (%) | 5,257 (92.2) | 448 (7.8) | <0.05 | 2,671 (93.8) | 175 (6.2) | <0.01 | 2,586 (90.5) | 273 (9.5) | <0.05 |
| Ethnicity, *n* (%) |  |  | <0.001 |  |  | <0.05 |  |  | <0.001 |
| White | 6,077 (92.3) | 507 (7.7) |  | 3,061 (93.7) | 2061(6.3) |  | 3,016 (90.9) | 301 (9.1) |  |
| Black | 832 (92.2) | 70 (7.8) |  | 418 (91.9) | 37 (8.1) |  | 414 (92.6) | 33 (7.4) |  |
| Others | 1,799 (88.9) | 225 (11.1) |  | 941 (91.1) | 92 (8.9) |  | 858 (86.6) | 133 (13.4) |  |
| Admission type, *n* (%) |  |  | <0.001 |  |  | <0.001 |  |  | <0.05 |
| Observatory & Elective | 1,624 (93.7) | 110 (6.3) |  | 811 (94.6) | 46 (5.4) |  | 813 (92.7) | 64 (7.3) |  |
| Emergent | 6,223 (91.8) | 559 (8.2) |  | 3,173 (93.5) | 221 (6.5) |  | 3,050 (90.0) | 338 (10.0) |  |
| Urgent | 861 (86.6) | 133 (13.4) |  | 436 (86.5) | 68 (13.5) |  | 425 (86.7) | 65 (13.3) |  |
| ICU type, *n* (%) |  |  | <0.001 |  |  | <0.001 |  |  | <0.001 |
| CVICU/CCU | 3,800 (94.3) | 230 (5.7) |  | 1,882 (96.1) | 76 (3.9) |  | 1,918 (92.6) | 154 (7.4) |  |
| TICU/SICU/Neurosurgical ICU | 2,589 (90.6) | 269 (9.4) |  | 1,384 (93.5) | 96 (6.5) |  | 1,205 (87.4) | 173 (12.6) |  |
| Medical/MedicalSurgical ICU | 2,319 (88.4) | 303 (11.6) |  | 1,154 (87.6) | 163 (12.4) |  | 1,165 (89.3) | 140 (10.7) |  |
| Severity of illness |  |  |  |  |  |  |  |  |  |
| SAPS II score | 34 (27-42) | 51 (40-61) | <0.001 | 34 (27-42) | 52 (41-63) | <0.001 | 34 (26-42) | 50 (40-61) | <0.001 |
| SOFA score (first day) | 4 (3-7) | 9 (6-12) | <0.001 | 4 (3-7) | 9 (6-12) | <0.001 | 4 (3-6) | 9 (6-12) | <0.001 |
| CCI | 5 (3-7) | 7 (5-9) | <0.001 | 5 (3-7) | 7 (5-9) | <0.001 | 5 (3-7) | 7 (5-9) | <0.001 |
| Sepsis-3 (fist day) *n* (%) | 4,939 (88.6) | 637 (11.4) | <0.001 | 2523 (90.2) | 274 (9.8) | <0.001 | 2416 (86.9) | 363 (13.1) | <0.001 |
| Initial lactate level(mmol/L) | 1.7 (1.2-2.5) | 2.5 (1.6-4.5) | <0.001 | 1.7 (1.2-2.5) | 2.5 (1.6-4.6) | <0.001 | 1.7 (1.2-2.6) | 2.5 (1.6-4.5) | <0.001 |
| Interventions (first day) |  |  |  |  |  |  |  |  |  |
| Mechanical ventilation , *n* (%) | 3,852 ( 88.5) | 502 (11.5) | <0.001 | 1,985 (91.0) | 196 (9.0) | <0.001 | 1,867 (85.9) | 306 (14.1) | <0.001 |
| Vasopressors, *n* (%) | 1,670 (81.7) | 373 (18.3) | <0.001 | 879 (85.2) | 153 (14.8) | <0.001 | 791 (78.2) | 220 (21.8) | <0.001 |
| RRT, *n* (%) | 235 (74.6) | 80 (25.4) | <0.001 | 109 (69.9) | 47 (30.1) | <0.001 | 126 (79.3) | 33 (20.7) | <0.001 |
| Vital Signs (first day) |  |  |  |  |  |  |  |  |  |
| Heart rate (bpm) | 83 (75-94) | 89 (78-102) | <0.001 | 84 (75-94) | 91 (80-104) | <0.001 | 83 (74-94) | 87 (76-101) | <0.001 |
| MAP (mmHg) | 76 (71-83) | 73 (67-80) | <0.001 | 76 (71-83) | 71 (66-78) | <0.001 | 76 (71-83) | 73 (68-81) | <0.001 |
| Respiratory rate (/min) | 18 (16-20) | 20 (18-24) | <0.001 | 18 (16-20) | 21 (18-25) | <0.001 | 18 (16-20) | 20 (18-23) | <0.001 |
| Temperature (C°) | 36.8 (36.6-37.1) | 36.7 (36.3-37.0) | <0.001 | 36.8 (36.6-37.1) | 36.7 (36.3-37.0) | <0.001 | 36.8 (36.6-37.1) | 36.7 (36.3-37.1) | <0.001 |
| SpO_2_(%) | 98 (96-99) | 97 (95-99) | <0.001 | 98 (96-99) | 97 (95-98) | <0.001 | 98 (96-99) | 97 (95-99) | <0.05 |
| Laboratory data (first day) |  |  |  |  |  |  |  |  |  |
| Hemoglobin (g/dL) | 10.6 (9.3-11.8) | 10.0 (8.8-11.6) | <0.001 | 10.6 (9.4-11.8) | 10.0 (8.9-11.5) | <0.001 | 10.6 (9.3-11.9) | 9.9 (8.8-11.7) | <0.001 |
| Hematocrit (%) | 31.7 (28.3-35.5) | 30.9 (27.2-35.4) | <0.001 | 31.7 (28.4-35.3) | 31.0 (27.8-35.1) | <0.05 | 31.7 (28.1-35.7) | 30.8 (27.0-35.6) | <0.05 |
| WBC (×10^3^/mm^3^) | 11.5 (8.6-15.1) | 12.5 (8.8-17.4) | <0.001 | 11.8 (8.8-15.3) | 11.9 (8.1-17.2) | 0.261 | 11.3 (8.4-14.8) | 12.8 (9.1-17.4) | <0.001 |
| Platelet (×10^3^/μL) | 175 (132-233) | 178 (117-243) | 0.240 | 174 (132-232) | 182 (116-255) | 0.685 | 178 (132-235) | 176 (117-241) | 0.197 |
| Sodium (mEq/L) | 139 (136-141) | 139 (135-142) | 0.786 | 139 (137-141) | 138 (134-141) | <0.05 | 139 (136-141) | 139 (136-142) | 0.065 |
| Chloride (mEq/L) | 106 (102-108) | 105 (100-108) | <0.001 | 106 (103-108) | 103 (99-108) | <0.001 | 106 (102-109) | 105 (101-109) | 0.065 |
| Potassium (mEq/L) | 4.2 (3.9-4.5) | 4.3 (3.9-4.8) | <0.001 | 4.2 (3.9-4.5) | 4.3 (3.9-4.9) | <0.001 | 4.2 (3.9-4.6) | 4.3 (3.9-4.8) | <0.001 |

CVICU: cardiovascular ICU, CCU: coronary care unit, TICU: trauma ICU, SICU: surgical ICU, CCI: Charlson comorbidity index, RPT: renal replacement therapy.

All values are expressed as n(%) or median(IQR). All values of 1^st^ day vital signs and laboratory data are averaged.
