# Supplementary material for: Impact on outcomes of measuring lactates prior to ICU in unselected heterogeneous critically ill patients: A propensity score analysis
Source: PLoS One. 2022 Nov 28;17(11):e0277948. doi: 10.1371/journal.pone.0277948 (PMC9704607; doi:10.1371/journal.pone.0277948)
Supplement: S3 Table — Direct and indirect effects are derived from when age, CCI, ICU severity scores, and initial lactate level are fixed at the mean values. Mechanical ventilation includes non-invasive ventilation. Vasopressors include norepinephrine, epinephrine, vasopressin, dobutamine, dopamine, and phenylephrine. (DOCX) [file pone.0277948.s004.docx]

**Table S3. Causal mediation analysis for therapeutic intervention in the PSM cohort**

| Therapeutic intervention | Direct effect (95%CI) | *Z* value | | | *p* value | | | Indirect effect (95%CI) | | | *Z* value | | | *p* value | | |
| --- | --- | --- | --- | --- | --- | --- | --- | --- | --- | --- | --- | --- | --- | --- | --- | --- |
| Mechanical ventilation | | | | | | | | | | | | | | | | |
| Number (n) | 0.64132 (0.15509-0.63448) | | | 3.23 | | | <0.01 | | | 0.00062 (-0.00813-0.00936) | | | 0.14 | | 0.890 | |
| Minutes to Ventilation | 0.76712 (0.37140-1.12798) | | | 3.88 | | | <0.001 | | | 0.00472 (-0.00758-0.01701) | | | 0.75 | | 0.452 | |
| Vasopressors | | | | | | | | | | | | | | | | |
| Number (n) | 0.48184 (0.20634-0.75652) | 2.86 | | | <0.01 | | | 0.00036 (-0.00139-0.00210) | | | 0.40 | | | 0.689 | | |
| Minutes to vasopressor | 0.53031 (0.11555-0.93900) | -2.52 | | | <0.05 | | | -0.00172 (-0.03430-0.03086) | | | -0.10 | | | 0.918 | | |
| RRT | | | | | | | | | | | | | | | | |
| Number (n) | -0.80137(-1.72062-0.11787) | | -1.71 | | | 0.088 | | | 0.00074 (-0.00259-0.00408) | | | 0.44 | | | 0.662 | |
| Minutes to RRT | -0.41908 (-0.80201 to -0.11232) | | -2.39 | | | <0.05 | | | 0.02816 (-0.05404-0.11037) | | | 0.67 | | | 0.502 | |
| A-line | | | | | | | | | | | | | | | | |
| Number (n) | 0.71007 (0.43979-0.98036) | | 5.15 | | | | <0.001 | | | 0.19879 (0.14061-0.25697) | | | 6.70 | | <0.001 | |
| Minutes to A-line | 1.10817 (0.62331-1.59301) | | 4.60 | | | | <0.001 | | | 0.07714 (0.22600-0.13168) | | | 2.77 | | <0.01 | |
| CV-line | | | | | | | | | | | | | | | | |
| Number (n) | 0.45602 (0.21863-0.94539) | | 3.46 | | | | <0.01 | | | -0.00034 (-0.00291-0.00222) | | | -0.26 | | 0.794 | |
| Minutes to CV-line | 0.60046 (0.21824-0.98268) | | 3.08 | | | | <0.01 | | | -0.00037 (-0.00393-0.00319) | | | -0.20 | | 0.839 | |
| PA-catheter | | | | | | | | | | | | | | | | |
| Number (n) | 0.38058 (0.14780-0.61336) | | 3.20 | | | | <0.01 | | | 0.05614 (0.04088-0.07140) | | | 7.21 | <0.001 | | |
| Minutes to PA-catheter | 2.36483 (0.30839-4.42127) | | 2.50 | | | | <0.05 | | | 0.11778 (-0.12795-0.36355) | | | 0.94 | 0.347 | | |
| Administration of IV antibiotics | | | | | | | | | | | | | | | | |
| Number (n) | 0.39967 (0.16161-0.63773) | | 3.29 | | | | <0.01 | | | 0.05097 (0.00957-0.09237) | | | 2.41 | 0.061 | |  |
| Minutes to IV antibiotics | 0.40351 (0.13339-0.67363) | | 2.93 | | | | <0.01 | | | -0.00495 (-0.04864-0.03875) | | | -0.22 | 0.824 | |  |
| Bolus fluid infusion | | | | | | | | | | | | | | | | |
| Number | 0.46651 (0.22045-0.71258) | | 3.72 | | | | <0.001 | | | 0.02193 (0.00303-0.04083) | | | 2.27 | <0.05 | |  |
| Minutes to bolus | 0.56915 (0.22248-0.91582) | | 3.11 | | | | <0.01 | | | 0.02333 (-0.00407-0.05053) | | | 1.67 | 0.095 | |  |

Direct and indirect effects are derived from when age, CCI, ICU severity scores, and initial lactate level are fixed at the mean values. Mechanical

ventilation includes non-invasive ventilation. Vasopressors include norepinephrine, epinephrine, vasopressin, dobutamine, dopamine, and　phenylephrine.
